# Video Game Use, Aggression, and Social Impairment in Adolescents with Autism Spectrum Disorder

**DOI:** 10.1007/s10803-022-05649-1

**Published:** 2022-07-12

**Authors:** Konnor Davis, Ana-Maria Iosif, Christine Wu Nordahl, Marjorie Solomon, Marie K. Krug

**Affiliations:** 1 Department of Psychiatry and Behavioral Sciences, University of California Davis, Sacramento, USA; 2 MIND Institute, University of California Davis, 2825 50th Street, Sacramento, CA 95817 USA; 3 Department of Public Health Sciences, University of California Davis, Sacramento, USA; 4 Imaging Research Center, University of California Davis, Sacramento, USA

**Keywords:** Autism spectrum disorder, Video games, Adolescence, Aggression, Social impairment

## Abstract

We used parent report data to investigate video game playing, aggression, and social impairment in adolescents with autism spectrum disorder. Parents of autistic adolescents were more likely to report that their child plays video games as a hobby compared to parents of adolescents with typical development and also reported that their children spent more time playing video games. For autistic participants, we found no differences in aggression levels or social impairment when comparing players versus non-players. However, playing video games “more than average,” as compared to “average” was associated with greater aggression and greater social impairment on “awareness” and “mannerisms” subscales. Future studies should focus on how type of video game(s) played is associated with these clinically important variables.

**Supplementary Information:**

The online version contains supplementary material available at 10.1007/s10803-022-05649-1.

In the twenty-first century, and especially in the United States (US), video games are ubiquitous. There are nearly 227 million total video game players in the US, with 51% playing over 7 h per week (2021 Essential Facts About the Video Game Industry, 2021). Seventy-six percent of American kids under 18 are classified as video game players (2021 Essential Facts About the Video Game Industry, 2021) and 90% of teens age 13–17 report that they play some type of video game (Anderson & Jiang, 2018). Eighty-three percent of teen girls report playing video games while 97% of teen boys report playing video games (Anderson & Jiang, 2018). A study of typically developing (TD) 6–12-year-olds showed that time spent playing video games averaged 1.3 h per day (Swing et al., 2010), and boys play for a significantly longer amount of time compared to girls (Gentile, 2009; Marshall et al., 2006).

Extensive research endeavors have investigated the positives and negatives of video game playing on youth. The negatives are heavily publicized and consist of warnings of addiction (Griffiths & Meredith, 2009), decreased social interaction (Anderson et al., 2010), and potential complex internal psychological processes that can produce increases in violence and aggression in players (Granic et al., 2014). On the other hand, video games can provide benefits in the social, emotional, motivational, and cognitive domains (Granic et al., 2014).

One of the most controversial topics in video game research is the heavily debated link between video games (specifically violent video games) and aggression and violence. An early study (Anderson & Dill, 2000) and a large meta-analysis (Anderson et al., 2010) concluded that “the evidence strongly suggests that exposure to violent video games is a causal risk factor for increased aggressive behavior, aggressive cognition, and aggressive affect and for decreased empathy and prosocial behavior” (Anderson et al., 2010). However, several studies and meta-analyses have countered the claims in Anderson and Dill (2000) and Anderson et al. (2010) (e.g., Ferguson, 2011, 2015; Ferguson et al., 2008). In addition, a recently published 10-year longitudinal study confirms no link between playing violent video games as early as 10 years old and aggressive behavior later in life (Coyne & Stockdale, 2021), and an American Psychiatric Association [APA] meta-analysis (2015) found no link between violent video games and aggression (Ferguson et al., 2020).

In recent years, there has been an increase in the empirical study of video game play in autistic youth. As a whole, studies have shown that children with autism spectrum disorder (ASD) spend a large amount of time playing video games (Mazurek et al., 2011; Orsmond & Kuo, 2011; Shane & Albert, 2008), have difficulty stopping video game sessions (Nally et al., 2000), and are more likely to exhibit video game addiction or preoccupation than their TD peers (Mazurek & Wenstrup, 2012). Mazurek and Wenstrup (2012) reported that children with ASD played video games an average of 2.0 h per weekday (2.4 h for males; 1.8 h for females) compared to the 1.3 h for TD (1.6 h for males; 0.8 h for females). Similarly, 41.4% of autistic adolescents chose to spend most of their free time playing video games, as compared to 18% of their TD peers (Mazurek et al., 2011). Increased playing time for children with either ASD or TD can lead to problems, including an umbrella of issues referred to as *problematic video game behavior* (Griffiths & Davies, 2005). Problematic video game behavior is of high clinical relevance due to its association with symptoms such as depression, anxiety, social isolation, fatigue, poor academic performance, and even video game addiction (Craig et al., 2021). Importantly, studies have shown that problematic video game behavior is common in individuals with neurodevelopmental disorders (Andreassen et al., 2016; González-Bueso et al., 2018). Core characteristics of ASD, such as impairment in social and communication skills and engagement in restricted and repetitive behaviors, can be associated with problematic video game playing (Engelhardt et al., 2013; Mazurek & Engelhardt, 2013). Lastly, it has been well documented that children with ASD are often targets of bullying behaviors, with almost 50% of autistic children reporting being bullied (Maïano et al., 2015). Although it is not as well-documented, children with ASD may also act as bullying perpetrators. In Maïano et al., 2015’s meta-analysis, (17 studies, 5000 + participants with ASD) they revealed that general school bullying perpetration (physical, verbal, and/or relational) is estimated to occur in 10% of school-aged autistic youth, which is similar to the percentage of TD peers who exhibit bullying behaviors. With an ever-increasing population of children playing video games, there is concern that cyber-bullying, which already occurs fairly frequently via video games (Patchin, 2018), will also affect an already vulnerable population of children as either victims, perpetrators, or victim-perpetrators. To date there is a lack of research on the rates of cyberbullying in video games specifically for autistic youth.

There are also benefits of playing video games, both for players with autism and with TD (Granic et al., 2014; Wiederhold, 2021). For children with TD, video games lead to positive interactions such as making new friends, competitive fun, or having an instant icebreaker (Kutner & Olson, 2011). For youth with ASD, video games (as an extension of online, asynchronous communication) may increase social functioning by offering opportunities for social interaction with decreased requirements for reading nonverbal cues and facial expressions, and for interpreting gestures (Walther, 2007). Additional research is needed to investigate whether the specific benefits of video game playing found in TD extend to ASD, and vice-versa.

Aggression and social impairment are two areas of research that are pertinent to core characteristics of ASD and the overall body of video game research. Aggression is a broad term that can incorporate many feelings and behaviors, such as arguing, screaming, destroying property, threatening, fighting, or attacking others (Mazurek et al., 2013). In addition to behaviors typically associated with aggression, children with ASD can present with other maladaptive behaviors such as self-injury, tantrums, and irritability (Erickson et al., 2016). Dominick et al. (2007) reported that 32.8% of school-age children with ASD display aggressive behaviors. On the other hand, a large-scale study of 1380 children and adolescents with ASD found that 56% were engaging in some form of aggressive action(s) at the time of assessment (Kanne & Mazurek, 2010). While research into aggression, and more specifically violence, associated with video gaming in TD samples is extensive, historically this has not been investigated as extensively ASD samples (Mazurek & Engelhardt, 2013). However, a recent study has shown that video game use is *not* related to negative behaviors in ASD (Alkhayat & Ibrahim, 2020). In Alkhayat and Ibrahim (2020), there was no correlation between duration of video game playing and negative behaviors associated with video game playing (such as isolation, poor school performance, playing games past bedtime, and agitation) for autistic children. While this study assessed negative behaviors more generally, the questionnaire assessing negative behaviors did query “Does your child beat, scream, or get angry if someone interrupts him/her while playing electronic games?”

Impairments in social interaction and communication are among the core diagnostic criteria for ASD according to the Diagnostic and Statistical Manual of Mental Disorders, 5th Edition (DSM-5; APA, 2013). A common misconception in the world of video games, especially those created decades ago when online communication was not as ubiquitous, is that players are socially isolated and spend most of their time alone when gaming, thereby impairing their social functioning (Lenhart et al., 2008). This idea that video games can exacerbate impairments in social functioning is common when considering youth with ASD. However, there is a growing body of research that supports the social benefits of gaming in TD, in terms of playing games with friends, playing competitively or cooperatively, and engaging in massive online communities for popular games that require teamwork and (virtual) communication (Granic et al., 2014). Ultimately, there is considerable evidence for both pros and cons of video games in respect to social aspects in TD, but there has been little research looking at the specific effects of video game playing on social impairment in ASD. However, Alkhayat and Ibrahim (2020) recently showed that families playing video games with their children, using video games to calm children, and having parental rules about which video games can be played, were factors associated with positive behaviors in relation to video game playing, such as improved communication and social skills and appropriate behaviors.

The objective of the current study was threefold: First, we compared video game playing status (whether participants played or not) and video game playing amount in adolescent participants with ASD and a non-autistic control group (TD) matched on FSIQ and sex. We also investigated video game playing status and amount in our ASD and TD groups while accounting for sex. Second, we examined the association between video game play and aggression within individuals with ASD. Finally, we investigated the association between video game playing and social impairment in adolescents with ASD. Based on previous research, we expected our cohort of autistic participants to spend more time playing video games than their TD peers. We anticipated that aggression would not be elevated in adolescents with ASD who are avid video game players due to recent research showing that aggression is not associated with video game playing in TD. Lastly, in light of research suggesting that video games can have a positive social benefit in those without ASD (Granic et al., 2014), we hypothesized that video game playing would be associated with lower scores on a measure of social impairment in our already impacted autistic adolescents.

## Methods

### Participants

Participants were enrolled in either of two longitudinal studies: the Cognitive Control in Autism (CoCoA) study or the Autism Phenome Project (APP) study conducted at the University of California (UC) Davis MIND Institute. Both studies had protocols that included the same assessments for hobbies, aggressive symptoms, and social functioning (see below for a detailed description of measures). To qualify for either study, autistic participants had to have a clinical best estimate diagnosis of autism which included presenting with a community diagnosis of ASD, and meeting criteria for autism on the Autism Diagnostic Observation Schedule—Second Edition (ADOS-2; Lord et al., 2000, 2012), which was administered by a research-reliable licensed clinical psychologist at the UC Davis MIND Institute, as well as meet other study-specific criteria detailed below. Both studies also included a typically developing, non-autistic, control group that was screened for autism before enrollment.

For the CoCoA study, autistic participants also needed to meet criteria on the Diagnostic and Statistical Manual (DSM-5) Checklist (APA, 2013) for ASD. The Social Communication Questionnaire—Lifetime Edition (SCQ; Rutter et al., 2003) was used as a screen for ASD in the potential TD participants. Given that a total score of ≥ 15 on the SCQ is consistent with a diagnosis of ASD, all participants in the TD group were required to have an SCQ total score of ≤ 11, which is consistent with *not* having ASD (Berument et al., 1999; Rutter et al., 2003). In addition, TDs in the CoCoA study exhibited no social communication disorders on a DSM-5 based symptom checklist and had no first-degree relatives with ASD. Lastly, in the CoCoA study, both autistic and TD individuals were required to have a Wechsler Abbreviated Scale of Intelligence—Second Edition (WASI-II; Wechsler, 1999, 2011) Full Scale Intelligence Quotient (FSIQ) ≥ 70, no other reported neurodevelopmental disorders (except for ADHD in ASD participants), and no history of seizure disorders. TD participants had no parent-reported Axis 1 psychopathology. Because the CoCoA study included MRI scanning, TD and ASD participants could only be enrolled if they were not taking psychotropic medications (with the exception of ADHD medications in ASD participants). ASD participants could enroll if they had comorbid psychiatric disorders (such as anxiety or depression), provided they were not taking psychotropic medications. Data from CoCoA Timepoint 1 (T1) for participants aged 12–17 years have been included in the presented analyses because data on video game playing were collected through a questionnaire designed for those ≤ 17 years of age.

For the APP study, inclusion criteria were established when participants initially enrolled in the study at 2–3.5 years of age (T1) and were based on the National Institutes of Health Collaborative Programs of Excellence in Autism diagnostic standards. They were then reassessed approximately one year after T1 (T2), two years after T2 (T3), and at ages 9–12 (T4). The current analyses use data from T4. Autistic participants were re-assessed with the ADOS-2 and SCQ (Rutter et al., 2003) at T4 to ensure that they continued to meet diagnostic criteria for ASD. Control participants are non-autistic children who were screened using the SCQ (for scores ≤ 11) at each timepoint and did not have a diagnosis of Intellectual Disability, Pervasive Developmental Disorder, or Specific Language Impairment. Both ASD and TD children had English as their primary language, resided with at least one biological parent, and were not diagnosed with any motor, vision, hearing, or other chronic health issue that would limit study participation. Full inclusion/exclusion criteria for APP are detailed in Kerns et al. (2021). All diagnoses (ASD/TD) were reaffirmed via testing at T4. The APP sample includes a wide range of developmental and cognitive profiles. Due to this being a case-matched study sample (see below), many of the individuals in the APP study with lower IQs, including some with intellectual disability, were not included in the main analyses.

Both the CoCoA and APP studies were approved by the UC Davis Institutional Review Board. Informed consent was obtained from the parent or guardian of each participant, and each participant provided assent if capable.

A total of 151 ASD (109 APP, 42 CoCoA) and 114 (70 APP, 44 CoCoA) TD participants met inclusion criteria. Two additional participants (ASD) were removed because their data was collected during the COVID-19 pandemic, which could affect video gaming habits. Data from all remaining participants was collected prior to the onset of the COVID-19 pandemic. These participants were submitted to an automated greedy matching algorithm (http://bioinformaticstools.mayo.edu/research/gmatch/) to identify a 1:1 matched sample. The algorithm allowed us to set a maximum difference on FSIQ (7 points) and require an exact match on sex and study when selecting a match. The final sample included 76 ASD-TD matched pairs. Participant characteristics are provided in Table 1.

**Table 1:** Participant characteristics

	ASD (*n* = 76)	TD (*n* = 76)	Group comparison
Age (years)	13.1 *(2.2)*	13.1 *(2.2)*	*U* = 5700.50, *p* = 0.68
Sex (F, M)	16, 60	16, 60	N/A
WASI-II/DAS-II FSIQ	107.5 *(11.8)*	107.9 (*11.2*)	*t* (150) = .22, *p* = 0.83
WASI-II/DAS-II VIQ	105.2 *(14.0)*	109.6 *(12.5)*	*t* (150) = 2.05, *p* = 0.04
WASI-II/DAS-II NVIQ	108.7 *(13.9)*	106.4 *(12.4)*	*U* = 5582.50, *p* = 0.39
SCQ	22.1 *(6.4)*	2.4 *(2.7)*	*U* = 2954.50. *p* < 0.001
ADOS-2 CSS	7.2 *(2.0)*	N/A	N/A

Data summarized as mean (standard deviation). Groups were compared with Student’s two-sample t-test for normally distributed variables and Wilcoxon rank-sum test (same as Mann–Whitney U) otherwise. All statistical tests were two-tailed

*ASD* autism spectrum disorder, *TD* typical development, *WASI-II* Weschler Abbreviated Scale of Intelligence, *DAS-II* differential ability scales, *FSIQ* full-scale IQ (DAS FSIQ scale = General Conceptual Ability Composite), *VIQ* verbal IQ (WASI VIQ scale = Verbal Comprehension Index), *NVIQ* nonverbal IQ (DAS NVIQ scale = Special Nonverbal Composite, WASI NVIQ scale = Perceptual Reasoning Index), *SCQ* social communication questionnaire, *ADOS-2* Autism Diagnostic Observation Schedule – 2nd edition, *CSS* calibrated severity score

One hundred and eleven participants (73 ASD; 38 TD) were removed from the sample after case matching. Half of the removed TD participants had an FSIQ above 120, while the majority of the ASD participants removed had FSIQs below 90. About half of the ASD participants removed (*n* = 36) had an FSIQ < 70, and FSIQ could not be computed for an additional two participants; one due to a lack of comprehension during the IQ assessment, and another due to behavioral difficulties during the testing session. Children with intellectual disability, which is diagnosed when there are deficits in intellectual functioning (FSIQ < 70) and adaptive behavior (APA, 2013), play video games less compared to ASD and TD children without intellectual disability (Mazurek et al., 2011; Rodríguez Jiménez et al., 2015). In a separate analysis we compared our ASD participants with FSIQ < 70 to our ASD participants with FSIQ ≥ 70 (for participant characteristics see Supplemental Table 1).

### Measures

#### ADOS-2 & SCQ (Autism Screening)

ASD symptoms and severity were evaluated using two gold-standard diagnostic assessments/interviews. First, the semi-structured standardized observation, the ADOS-2 (Lord et al., 2000, 2012), was administered by licensed clinicians at the UC Davis MIND Institute, yielding a total score and a Calibrated Severity Score (CSS; Gotham et al., 2009) which was used to compare scores across modules (with a CSS ≥ 4 necessary for ASD). The SCQ (Rutter et al., 2003), which is a 40 question, dichotomous, behavioral checklist that can be used as a proxy for autism characteristics (Adams et al., 2019; Westerveld et al., 2017) was administered to all participants.

#### WASI-II & DAS-II

The CoCoA study used the WASI-II to measure intellectual ability (Weschler, 2011). After completing the four subtests, FSIQ, verbal comprehension (verbal IQ), and perceptual reasoning (nonverbal IQ) scores were produced. The APP study used the Differential Ability Scales – Second Edition (DAS-II; Elliot, 2007) as their standard assessment for measuring intelligence. The General Conceptual Ability (GCA) composite was reported as a measure of FSIQ, verbal IQ composite was reported simply as verbal IQ, and the Special Nonverbal Composite (SNC) was used for nonverbal IQ.

#### CBCL

The Child Behavior Checklist/6–18 years (CBCL; Achenbach & Rescorla, 2001), is a commonly used parent report questionnaire including a range of problematic behaviors and emotions that map to eight syndrome scales and six DSM-oriented scales. The CBCL Aggressive Behavior Syndrome Scale is a compilation of 18 individual item scores/responses and was used to assess aggression. Examples include physical aggression, teasing, threatening, noncompliance, and destruction of items. The CBCL provides an age and gender adjusted T-Score, percentile, and a qualitative description (normal, borderline clinical, clinical). T-Scores ≥ 70 indicate clinical significance. Due to very low levels of aggression in the TD group (61% with an average or below average T-score; 3% with a borderline clinical T-score, and 0% with a clinical range T-score), aggression analyses were restricted to the ASD group, which had a broader range of Aggression T-scores (only 27% with an average or below average T-score; 13% with a borderline clinical T-score and 3% with a clinical range T-score). Table 2 includes the CBCL Aggression mean T-Score for each diagnostic group.

**Table 2:** CBCL (aggression) and SRS-2 (social impairment) for ASD and TD participants

	ASD (*n* = 76)	TD (*n* = 76)	Group comparison
CBCL Aggression T-Score^a^	56.7 *(6.7)*	52.2 *(4.2)*	*U* = 6950.50, *p* < 0.001
SRS-2 Awareness T-Score^b^	69.4 *(13.5)*	46.2 *(8.3)*	*U* = 7249.50, *p* < 0.001
SRS-2 Cognition T-Score^c^	66.8 *(12.3)*	44.1 *(6.1)*	*U* = 7749.00, *p* < 0.001
SRS-2 Communication T-Score^d^	69.9 *(12.4)*	45.1 *(6.9)*	*U* = 7694.50, *p* < 0.001
SRS-2 Motivation T-Score^e^	67.0 *(12.5)*	46.0 *(6.5)*	*U* = 7333.50, *p* < 0.001
SRS-2 Mannerisms T-Score^f^	71.5 *(12.9)*	46.0 *(5.4)*	*U* = 7868.00, *p* < 0.001
SRS-2 Total T-Score^g^	71.4 *(12.2)*	44.9 *(6.3)*	*U* = 7145.50, *p* < 0.001

Scores are summarized as mean (standard deviation). All scores were not normally distributed, so Wilcoxon rank-sum test (same as Mann–Whitney U) was used to assess group differences. All statistical tests were two-tailed

*ASD* autism spectrum disorder, *TD* typical development, *CBCL* Child Behavior Checklist, *SRS-2* Social Responsiveness Scale-2nd edition, *Mannerisms* restricted, repetitive behavior subscale

Data missing for: ^a^ One participant in ASD group

^b^Five participants in ASD group and 4 in TD

^c^Four participants in ASD group and 1 in TD

^d^Five participants in ASD group

^e^Six participants in ASD group and 2 in TD

^f^Four participants in ASD group

^g^Seven participants in ASD group and 5 in TD

The front page of the CBCL provides parents with a “hobbies” section that includes blank lines in which parents can write in up to three of their child’s hobbies (e.g., video games, dolls, reading, piano, etc.), or select none, based on an open-ended question as a prompt (“Please list your child’s favorite hobbies, activities, and games, other than sports.”). Participants were divided into “Player” and “Non-player” groups based on the written responses to the “hobbies” prompt. Our accepted responses that indicate video game players include simple phrases such as “video games” and “computer games,” or more specific indicators such as “Xbox” or “Fortnite.” More general responses such as “computer” or “iPad” were also accepted. However, if further details provided indicated that video games were not being played on the electronic device (such as “watching movies on the iPad”) then the participant was categorized as a “Non-player.” To evaluate whether including the players with more general responses had an impact on our findings, we conducted a sensitivity analysis by rerunning the matching algorithm and the main analyses after removing those participants.

Once a hobby is written in, there are four options to select from to answer the question: “Compared to others of the same age, about how much time does he/she spend in each?” The options are *Less Than Average*, *Average*, *More Than Average*, and *Don’t Know*. From these data we created four video game playing categories that reflect the *amount* of time spent playing video games: *Video game not listed as a hobby (“don’t play”), play video games less than average (“less than average”), play video games an average amount of time (“average”),* and *play video games more than an average amount of time (“more than average”).* Due to the limited information gained from the “don’t know” category, individuals whose parents made this response (1 TD, 3 ASD) were excluded from analyses that investigated amount of video game playing, although they were included in analyses investigating players versus non-players.

#### SRS-2

Social functioning was assessed using the Social Responsiveness Scale—Second Edition (SRS-2; Constantino & Gruber, 2012). The SRS-2 is a 65-item parent report questionnaire to detect and quantify the severity of social impairment across the autism spectrum. Responses on the SRS-2 are used to calculate a total score and five treatment subscale scores (*M* = 50, SD = 10): Social Awareness, Social Cognition, Social Communication, Social Motivation, and Restricted Interests and Repetitive Behavior (also referred to as “Mannerisms”). Elevated T-Scores (T-Scores ≥ 60) indicate more clinically significant social difficulties (e.g., more ASD symptoms endorsed) within the chosen domain. Out of 69 autistic participants with SRS-2 Total T-scores available (from our case matched sample), 55 had clinically significant social impairment (80%). Social impairment within the TD group was not analyzed due to the lack of the presence of this core symptom of ASD in most TD youth. Table 2 summarizes the SRS-2 subscale T-Scores based on diagnostic group.

### Statistical Methods

Differences in video game playing status between ASD and TD groups were assessed using chi-square tests. Mantel–Haenszel chi-square tests were used for amount of playing categories, to account for the ordinality in the data. Cochran–Mantel–Haenszel tests were used to examine differences in playing status and the amount of playing between ASD and TD groups while accounting for sex. Normality testing confirmed that aggression and social impairment data were not normally distributed, thus non-parametric tests (Wilcoxon rank-sum and Kruskal–Wallis) were employed to test differences in these scores. Analyses of aggression and social impairment scores across playing status and amount of playing categories were restricted to ASD participants, given the limited range of these variables in TD. For each aggression and social impairment outcome, significant overall differences among amount of playing categories were followed up with the Dwass, Steel, Critchlow-Fligner post-hoc procedure, which corrects for multiple comparisons, to determine which pairs of playing categories differed. All analyses were implemented using SAS Version 9.4 (SAS Institute Inc., Cary, NC). All tests were two-sided, and *p*-values < 0.05 were considered statistically significant.

## Results

### Video Game Playing

**Fig. 1:**
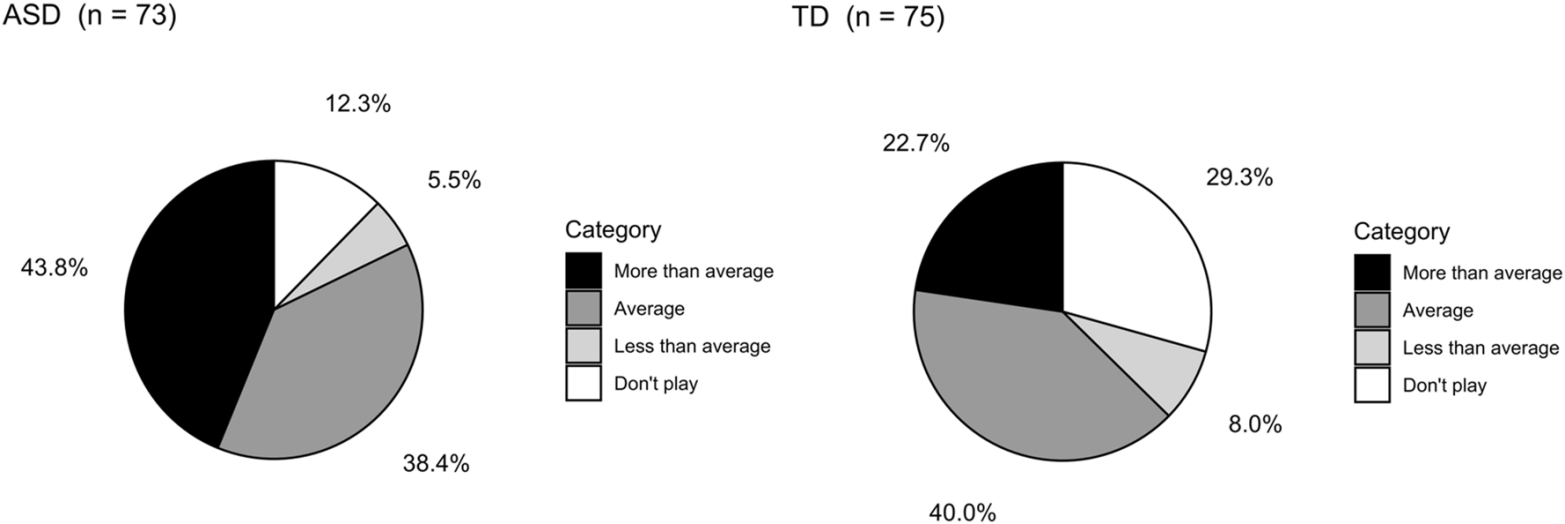
Amount of time spent playing video games, based on caregiver’s CBCL response, was greater for participants with ASD than participants with TD (Mantel–Haenszel ${\chi }^{2}$(1, *n* = 148) = 10.09, *p* = 0.002). *ASD* autism spectrum disorder, *TD* typical development, *CBCL* child behavior checklist

There was a significant difference in video game playing status (Player/Non-player) between diagnostic groups [χ^2^ (1, *n* = 152) = 6.80, *p* = 0.009], with parents of the autistic adolescents reporting video games as a hobby more frequently than those of TD adolescents. Similarly, significant diagnostic group (ASD/TD) differences in the amount of playing (don’t play, play less than average, play average, play more than average) were found [Mantel–Haenszel χ^2^ (1, *n* = 148) = 10.09, *p* = 0.002] (Fig. 1). These findings remained significant even when participants with more general electronic-device related hobbies (such as “iPad”) were excluded from the analyses.

To control for the potential difference in video game playing behavior between females and males, we redid our main analyses adjusting for sex. The results of the adjusted analyses confirmed the main results. After adjusting for sex, we found significant differences between ASD and TD in playing status (Player/Non-Player) [Cochran-Mantel–Haenszel χ^2^ (1, *n* = 152) = 8.17, *p* = 0.004] and amount of playing (don’t play, play less than average, play average, play more than average) [Cochran-Mantel–Haenszel χ^2^ (1, *n* = 148) = 12.21, *p* < 0.001]. Percentages for video game playing category, broken down by diagnostic group and sex, are shown in Supplemental Fig. 1.

Lastly, we found a significant difference between our FSIQ-matched ASD participants and the ASD participants with FSIQ < 70 (removed from the FSIQ-matched sample) and video game playing status group (Player/Non-player) [χ^2^ (1, *n* = 147) = 18.03, *p* < 0.001]. Parents of autistic individuals with FSIQ < 70 reported video games as a hobby less frequently than the parents of ASD participants with FSIQ of 70 or greater. There were also significant differences between the groups based on amount of playing (don’t play, play less than average, play average, play more than average), [Mantel–Haenszel χ^2^ (1, *n* = 141) = 15.68, *p* < 0.001] (Supplemental Fig. 2).

**Fig. 2:**
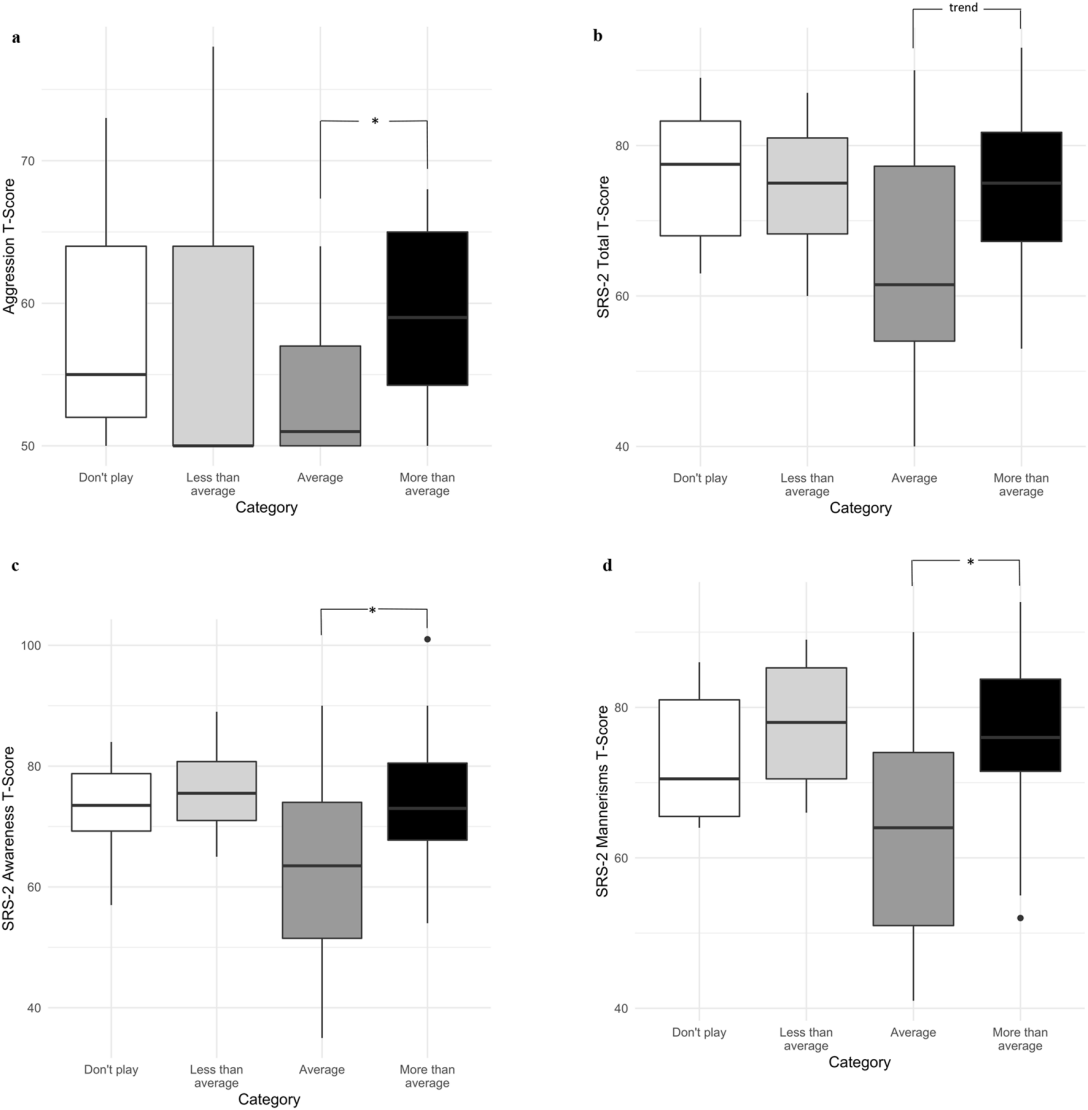
CBCL Aggression (*n* = 72) (**a**), SRS-2 Total (*n* = 66) (**b**), SRS-2 Awareness (*n* = 68) (**c**), and SRS-2 Mannerisms (*n* = 69) (**d**) in autistic adolescents when considering amount of video game playing category. Significant group differences among amount of playing categories were followed up with the Dwass, Steel, Critchlow-Fligner post-hoc procedure, which corrects for multiple comparisons, to determine which pairs of categories differed. ^*^p < 0.05 (corrected for multiple comparisons). *CBCL* child behavior checklist, *SRS-2* social responsiveness scale-2nd edition

### Aggressive Behavior

No significant differences were found in CBCL Aggression T-scores based on video game playing status (see Table 3). When using the amount of playing categories, CBCL Aggression T-scores were found to be significantly different [χ^2^ (3, *n* = 72) = 14.06, *p* = 0.003] (Table 4). Follow-up post-hoc non-parametric pairwise comparisons were used to confirm that the significant difference was between the “more than average” (*M* = 59.0) and “average” group (*M* = 53.3) (*z* = 3.79, *p* < 0.001). No other comparisons were significant (Fig. 2a).

**Table 3:** CBCL (aggression) and SRS-2 (social impairment) for ASD players and non-players

	Players (*n* = 67)	Non-players (*n* = 9)	Group comparison
CBCL Aggression T-Score ^a^	56.6 *(6.5)*	57.9 *(8.2)*	*U* = 377.00, *p* = 0.57
SRS-2 Awareness T-Score^b^	68.9 *(13.9)*	73.0 *(8.7)*	*U* = 335.00, *p* = 0.40
SRS-2 Cognition T-Score^c^	66.1 *(12.6)*	72.8 *(7.5)*	*U* = 375.50, *p* = 0.14
SRS-2 Communication T-Score^bd^	69.3 *(12.6)*	74.8 *(10.7)*	*U* = 352.00, *p* = 0.25
SRS-2 Motivation T-Score^d^	66.2 *(12.1)*	72.9 *(15.0)*	*U* = 358.50, *p* = 0.17
SRS-2 Mannerisms T-Score^c^	71.3 *(13.4)*	73.1 *(9.1)*	*U* = 299.00, *p* = 0.91
SRS-2 Total T-Score^e^	70.8 *(12.4)*	76.5 *(9.8)*	*U* = 345.00, *p* = 0.23

Scores are summarized as mean (standard deviation). All scores were not normally distributed, so Wilcoxon rank-sum test (same as Mann–Whitney U) was used to assess group differences. All statistical tests were two-tailed

*ASD* autism spectrum disorder, *CBCL* child behavior checklist, *SRS-2* Social Responsiveness Scale-2nd edition, *Mannerisms* restricted, repetitive behavior subscale

Data missing for: ^a^1 participant in players group

^b^4 participants in players group and 1 in non-players

^c^3 participants in players group and 1 in non-players

^d^5 participants in players group and 1 in non-players

^e^6 participants in players group and 1 in non-players

**Table 4:** CBCL (aggression) and SRS-2 (social impairment) for ASD participants by video game playing category

	Don’t play (*n* = 9)	Less than average (*n* = 4)	Average (*n* = 28)	More than average (*n* = 32)	Group comparison
CBCL Aggression T-Score^a^	57.9 *(8.2)*	59.3 *(16.2)*	53.3 *(4.2)*	59.0 *(6.0)*	χ^2^ (3) = 14.06, *p* = 0.003
SRS-2T-Score					
Awareness^b^	73.0 *(8.7)*	76.3 *(10.0)*	62.8 *(15.7)*	73.7 *(11.0)*	χ^2^ (3) = 8.57, *p* = 0.04
Cognition^c^	72.8 *(7.5)*	69.5 *(11.0)*	63.4 *(15.1)*	68.5 *(10.4)*	χ^2^ (3) = 4.22, *p* = 0.24
Communication^b^	74.8 *(10.7)*	71.5 *(15.8)*	64.9 *(13.8)*	72.1 *(10.5)*	χ^2^ (3) = 5.58, *p* = 0.13
Motivation^d^	72.9 *(15.0)*	64.5 *(9.4)*	63.0 *(12.3)*	68.6 *(11.9)*	χ^2^(3) = 4.34, *p* = 0.23
Mannerisms^c^	73.1 *(9.1)*	77.8 *(10.6)*	64.5 *(14.7)*	75.7 *(10.3)*	χ^2^(3) = 10.43, *p* = 0.02
Total^e^	76.5 *(9.8)*	74.3 *(11.5)*	65.2 *(14.1)*	74.4 *(10.2)*	χ^2^(3) = 7.87, *p* = 0.05

Scores are summarized as mean (standard deviation). All scores were not normally distributed; therefore, Kruskal–Wallis test was used to assess group differences. All statistical tests were two-tailed. Significant group differences between amount of playing categories were followed up with the Dwass, Steel, Critchlow-Fligner post-hoc procedure, which corrects for multiple comparisons, to determine which pairs of playing categories differed

*ASD* autism spectrum disorder, *CBCL* child behavior checklist, *SRS-2* Social Responsiveness Scale-2nd edition, *Mannerisms* restricted, repetitive behaviors subscale

Data Missing for: ^a^1 participant in the less than average group

^b^1 participant in the don’t play group, 2 participants in the average group, 2 participants in the more than average group

^c^1 participant in the don’t play group, 1 participant in the average group, 2 participants in the more than average group

^d^1 participant in the don’t play group, 3 participants in the average group, 2 participants in the more than average group

^e^1 participant in the don’t play group, 4 participants in the average group, 2 participants in the more than average group

### Social Impairment

No significant differences were found in SRS-2 Total T-Score for video game playing status. As presented in Table 3, Wilcoxon rank-sum tests for each of the five SRS-2 subscales comparative to video game playing status, were not significant. When considering the four categories for amount of playing, three of the five SRS-2 subscales were not significant (Cognition, Communication, Motivation) (Table 4). The SRS-2 Total T-Score was significantly different across groups [χ^2^ (3, *n* = 66) = 7.87, *p* = 0.05], as were the SRS-2 Awareness [χ^2^ (3, *n* = 68) = 8.57, *p* = 0.04] and Mannerisms [χ^2^ (3, *n* = 69) = 10.43, *p* = 0.02] subscales (Table 4). Post-hoc non-parametric pairwise comparisons confirmed that the significant difference for the Awareness subscale was between the “play more than average” (*M* = 73.7) and “play average” group (*M* = 62.8) (*z* = 2.64, *p* = 0.04) (Fig. 2c). For the Mannerisms subscale the difference was also significant between the “play more than average” (*M* = 75.7) and “play average” group (*M* = 64.5) (*z* = 3.01, *p* = 0.01) (Fig. 2d). For SRS-2 Total, post-hoc pairwise comparisons were not significant, although the difference between “play more than average” (*M* = 74.4) and “average” (*M* = 65.2) had a trend-level *p*-value (*z* = 2.45, *p* = 0.07) (Fig. 2b).

## Discussion

The current study investigated video game playing status (player vs. non-player) and amount of time spent playing video games (don’t play, less than average, average, more than average) in adolescents with ASD and TD, and also examined the association between video game playing and aggression and social impairment in adolescents with ASD. Parents of autistic participants reported their children as having video games as a hobby more often than parents of TD children. The analysis looking at amount of time spent playing video games also showed that ASD and TD differed significantly, with autistic adolescents more often falling into the “play more than average” category compared to TD (Fig. 1). Both of these findings remained significant when controlling for sex. Our data show that video game playing status is not associated with aggression in ASD. However, when considering the four playing amount categories, there was a significant difference driven by autistic adolescents whose parents report that they are playing more than average (versus an average amount of time) having greater aggression scores (Fig. 2a). There were no significant differences in SRS-2 Total T-Scores, or in any of the five SRS-2 subscales, when comparing ASD players versus non-players. When investigating the amount of playing categories for the SRS-2, significant differences were found for the Total T-score as well as two of the five SRS-2 subscales (Social Awareness, Mannerisms). For both subscales, the “play more than average” group showed greater social deficits than the “play average” group, and the same was found at a trend-level for the Total T-score (Fig. 2b–d).

Due to low numbers of female participants (*n* = 16 per group) interpretation of our data on video game playing in girls is limited. However, it was surprising to see that ASD vs. TD differences in video game playing status appeared to be even more pronounced in girls than boys (Supplemental Fig. 1). This was driven by our finding that a large portion of our TD girls (75%) were not classified as video game players, which differs from recent reports showing that video game playing, while lower in girls than boys, is still highly prevalent in girls (Anderson & Jiang, 2018). This could be due to the nature of our measure. Many of these girls classified as “non-players” may play video games, but perhaps not frequently enough for the parent to list it as a hobby. This interesting finding does warrant future investigations into video game playing habits in autistic and TD girls, to see whether differences in video game play are more prevalent in females.

The finding that a greater than average amount of playing time is associated with increased aggression is in discordance with Alkhayat and Ibrahim’s (2020) finding that duration of video game play is not associated with negative behaviors in autistic children. However, in Alkhayat and Ibrahim’s study, autistic and TD children did not differ in amount of time spent playing video games, which also differs from our findings, as well as other studies that have shown greater video game playing time in autistic populations (Mazurek & Wenstrup, 2012; Mazurek et al., 2011). Additionally, their negative behaviors variable encompassed many negative behaviors, and not aggression exclusively. Thus, there is a need for further research into aggression and video game playing in autism (Mazurek & Engelhardt, 2013). Future research could be supplemented with a video game specific questionnaire that asks parents to delineate not only the exact amount of time played, but what genres of games are being played and the content within the specific game. In much of the TD research, the genre of the video game is a contributing factor in the various published aggression findings (Anderson & Dill, 2000; Anderson et al., 2010), and it remains to be seen whether the same holds true for ASD.

The SRS-2 Restricted Interests and Repetitive Behavior (“Mannerisms”) subscale examines to what extent restricted, repetitive behaviors and interests are present. Video games often become a restricted interest in adolescents with ASD, as playing these games can morph from a leisurely hobby to an intense preoccupation. Thus, these findings agree with previous research showing that core symptoms of ASD, namely that the tendency to develop intense interests or encompassing preoccupations, plays a role in the extent of video game playing (Mazurek & Engelhardt, 2013). In short, restricted interests are “highly restricted, fixated interests that are abnormal in intensity or focus” (APA, 2013) *this needs a link to the reference whereas gaming addiction (also known as Gaming Disorder) requires at least 12 months of impaired control over gaming, increasing priority given to gaming over other activities, and continuation or escalation of gaming despite negative consequences (most often clinically significant distress/impairment in various areas of functioning) (Coutelle et al., 2021). Restricted interests are a core symptom of ASD while gaming addiction follows other addictions in utilizing a five-component model of behavioral addiction. Furthermore, Coutelle et al. (2021) suggest that high levels of video gaming in ASD can be explained by gaming addiction, but that the influence of restricted interests cannot be excluded. There is considerable evidence on either side of this debate (video gaming in ASD presenting as an addiction or a restricted interest) such that differentiating between the two is difficult and an ongoing clinical dilemma in which there has been a continuing evolution of perspectives about diagnosis and treatment. Regardless of whether higher scores on the Restricted Interested and Repetitive Behavior (Mannerisms) subscale in autistic children who play video games “more than average” represents a manifestation of the core symptoms of ASD or gaming addiction (or both), associations between video game play and this particular subscale are perhaps least surprising, and in isolation should not be interpreted as a more general association of video game playing and global social deficits.

The SRS-2 Awareness subscale refers to sensory aspects of reciprocal interactions and detection of social cues. Examples include awareness of what others are thinking/feeling and knowing when they are talking too loudly, to name a few. Social awareness and reciprocity are often impaired in children with ASD and this can be compounded by those who play a greater amount of video games. Those who play a greater amount of video games may be at risk for social isolation (Anderson et al., 2010; Griffiths & Davies, 2005) possibly due to decreased opportunities to have reciprocal social interactions because more time is devoted to gaming as compared to other activities that would stimulate reciprocal interactions. Thus, these findings agree with previous research. On the other hand, those who gravitate toward video games may do so because they are less inclined to engage in activities with higher social demands that may be challenging for them. As with the Mannerisms subscale finding, this finding also requires further research into the likely complex interaction between video game play and social functioning in autistic individuals. However, it is important to note that video gaming could have a positive effect on this subscale, under certain conditions. For example, one could imagine those who play more video games, specifically games in-person or online games requiring conversation, would have increased exposure to reciprocal interactions due to the nature of the games they are playing (Walther, 2007).

Our significant findings for amount of play categories were driven by significantly higher scores in the “play more than average” compared to the “play average” group, both for our measures of aggression and social impairment (Social Awareness and Mannerisms subscales). However, when considering all four video game playing categories, the means for “play more than average” are not particularly high, but rather the means for “play average” appear low (Fig. 2; Table 4). Thus, it may be that, overall, average video game play is associated with less aggression and less social deficits, as opposed to the reverse interpretation, that more than average amount of time spent playing video games is associated with high levels of aggression and greater social impairment. However, a firm conclusion is not possible given the lack of significance between the other post-hoc comparisons, as well as the low numbers of participants in the “don’t play” and “play less than average” categories.

It is important to note that our sample includes autistic children who also may have Attention Deficit Hyperactivity Disorder (ADHD), thus the relationship between ADHD, video game play, aggression, and social functioning should also be considered in light of our results. While some studies show that children with ADHD do not play video games more or less in comparison to either TD or autistic children (Bioulac et al., 2008; Mazurek & Engelhardt, 2013), a recent study of children aged 4–12 years showed that children with ADHD spend significantly more time playing video games than TD peers on both weekdays and weekends (Masi et al., 2021). Despite debate over playing amounts, children with ADHD exhibit more addictive/compulsive behaviors in relation to video games (Kietglaiwansiri & Chonchaiya, 2018; Masi et al., 2021). In regards to aggression, children with ADHD do show higher rates of proactive and reactive aggression (Slaughter et al., 2019). In Bioulac et al. (2008), a subgroup of ADHD participants who met criteria for problematic video game usage also had significantly higher aggression scores than ADHD participants without problematic video game usage or TD controls. It is possible that comorbid ADHD in our autistic participants may have contributed to our aggression findings. Lastly, like children with ASD, social problems are a prominent feature for children with ADHD (Kofler et al., 2011). However, there has been little research looking at the relationship between video game playing and social impairment in ADHD, thus ADHD-specific studies are needed to investigate possible associations.

One limitation to our study is that the CBCL is not a video game-specific questionnaire. It asks parents to list up to three hobbies that are not sports, and based on this open-ended question we classified participants as video-game players or non-players. However, it is uncertain how the format of this question impacted our classification. For example, for children with many hobbies, parents might not write in “video games” if other hobbies are more favored. These children would be classified as a non-players, when perhaps they were actually video-game players in the “less than average” category. A question specifically asking about video-game playing would have been ideal for accurate classification. As discussed above, the CBCL does not assess what type of video games are being played. Another limitation of the CBCL is that it does not assess quantitative playing time. Furthermore, the question used to assess amount of time spent playing asks parents to compare their child to others of the same age. When parents of autistic children answered that question, it is unknown whether they were comparing their child to other autistic children or TD children. Next, for the CoCoA sample, there were significant constraints imposed on initial study enrollment because use of psychotropic medications (other than ADHD medications) was an exclusion. This created a group of participants where baseline levels of certain comorbid conditions (anxiety, depression, aggression) were less prevalent than would be expected in a population type sample. Should levels of these conditions in our sample have matched a population type sample, there may have been further/larger group differences, specifically when looking at aggression. Lastly, it is important to note that this study used an FSIQ-matched sample. While this eliminated the confounding effects of IQ differences, it also means conclusions from our main analyses cannot be extended across the entire range of autistic individuals. Supplemental analyses showed that parents of autistic adolescents with lower (< 70) FSIQ were less likely to report video games as a hobby in comparison to parents of autistic adolescents with higher IQs (Supplemental Fig. 2). If they did play video games, adolescents with lower IQs typically played on a tablet or phone as opposed to traditional video games played using a gaming console.

In the future, further research using dedicated video game questionnaires is highly recommended. These questionnaires allow collection of in-depth information about when (weekday vs. weekend) and how often (typically in a quantitative fashion such as number of hours) games are being played. Secondly, these questionnaires also help in regard to determining what genres of games are being played, and with that information, more detailed conclusions about associations with aggression and social functioning can be made. Fortunately, a few popular video game questionnaires exist such as the Problem Video Game Playing Test (King et al., 2009) and the General Media Habits Questionnaire (Craig Alan Anderson et al., 2007; Gentile et al., 2004).

Video game specific questionnaires can also be enhanced by including general questions regarding aggression and how the adolescent feels when playing certain genres, especially for self-report questionnaires. For example, Gentile et al. (2004) adapted Anderson and Dill’s (2000) questionnaire to include questions about violence in the video games played. Different methods to specifically assess aggression in ASD may also be helpful, such as the Aberrant Behavior Checklist Irritability subscale (Aman & Singh, 1994), as well as computer tasks or games that measure responses to aggressive stimuli (Erickson et al., 2016), or having an evaluator observe aggressive behavior during experimental sessions (Erickson et al., 2016).

Lastly, while the bulk of current research has investigated negative aspects of video games (including social isolation), future research is needed to focus more on possible benefits of video gaming in ASD. What qualifies as a video game is largely open ended, and many games have a significant social component built into them (e.g., competitive play requires intense communication and teamwork in order to succeed). With the advent of the Internet and more sophisticated platforms to play video games that incorporate text or voice chat, it has never been easier to log on and play with others. One may assume that a child who is engaged in more communicative based video games is actively engaging in social skills through periods of increased communication with other peers in order to play the game, thus relationships between video game playing and social functioning may be highly dependent upon the type of game being played.

During the COVID-19 pandemic, video games have become a key outlet to avoid social isolation during long-haul stretches of lockdowns and quarantines. In a 2020 survey conducted by Google, 35% of American respondents said their decision to spend more time playing video games now than before the pandemic was to connect with friends and family (Google/Savatana, 2020). Furthermore, 59% of parents report using some form of educational video game for their children during the COVID-19 pandemic, and 63% of those parents report that the educational games were very or extremely effective (*2021 *Essential Facts About the Video Game Industry, 2021). While all data reported here was collected prior to the COVID-19 pandemic, future studies investigating time spent playing video games, as well as the amount of time spent playing video games for social communication or educational purposes, should consider possible associations with the COVID-19 pandemic and restrictions in place during the time these behaviors were assessed.

## Supplementary Information

Below is the link to the electronic supplementary material.

Supplementary file1 (DOCX 18194 kb)
