# Correction: Video Game Use, Aggression, and Social Impairment in Adolescents with Autism Spectrum Disorder

**DOI:** 10.1007/s10803-022-05694-w

**Published:** 2022-07-29

**Authors:** Konnor Davis, Ana-Maria Iosif, Christine Wu Nordahl, Marjorie Solomon, Marie K. Krug

**Affiliations:** 1 Department of Psychiatry and Behavioral Sciences, University of California Davis, Sacramento, USA; 2 MIND Institute, University of California Davis, 2825 50th Street, Sacramento, CA 95817 USA; 3 Department of Public Health Sciences, University of California Davis, Sacramento, USA; 4 Imaging Research Center, University of California Davis, Sacramento, USA

## Correction to: Journal of Autism and Developmental Disorders 10.1007/s10803-022-05649-1

The wrong Supplementary file was originally published with this article; it has now been replaced with the correct file.

The original article has been corrected.

## Supplementary Information

Below is the link to the electronic supplementary material.

Supplementary file1 (DOCX 18193 kb)

