# Supplementary material for: Video Game Use, Aggression, and Social Impairment in Adolescents with Autism Spectrum Disorder
Source: J Autism Dev Disord. 2022 Jul 12;53(9):3567–80. doi: 10.1007/s10803-022-05649-1 (PMC10465670; doi:10.1007/s10803-022-05649-1)
Supplement: Supplementary file 1 — Supplementary file1 (DOCX 18194 kb) [file 10803_2022_5649_MOESM1_ESM.docx]

**Supplemental Table 1** Demographic and clinical characteristics for ASD participants who were eligible for the study

|  | ASD,  FSIQ < 70  (*n* = 36) | ASD,  FSIQ ≥ 70  (*n* = 111) | Group Comparison |
| --- | --- | --- | --- |
| Age (years) | 11.6 *(1.1)* | 12.8 *(2.1)* | *U* = 2063.00, *p* = .007 |
| Sex (F, M) | 6, 30 | 24, 87 | *X ^2^*= .41, *p* = .52 |
| WASI-II/DAS-II FSIQ | 43.9 *(15.0)* | 100.7 (*17.0*) | *U* = 666.00, *p* < .001 |
| WASI-II/DAS-II VIQ^a^  WASI-II/DAS-II NVIQ^b^  SCQ^b^  ADOS-2 CSS | 33.4 *(12.3)*  49.7 *(17.5)*  26.5 *(5.0)*  8.1 *(1.5)* | 97.5 *(17.5)*  102.4 *(16.6)*  22.0 *(6.6)*  7.4 *(2.0)* | *U* = 675.00, *p <* .001  *U* = 672.00, *p <* .001  *t* (144) = 3.71, *p <* .001  *U* = 3022.00, *p =* .10 |

Note: Data summarized as mean (standard deviation). Groups were compared with Student’s t-test for normally distributed variables and Wilcoxon rank-sum test (same as Mann-Whitney U) otherwise. All statistical tests were two-tailed.

Data missing for: ^a^1 participant in FSIQ ≥ 70 group; ^b^1 participant in FSIQ ≥ 70 group and 1 participant in FSIQ < 70 group;

*ASD* Autism Spectrum Disorder, *FSIQ* Full-scale IQ (DAS FSIQ scale = General Conceptual Ability Composite), *WASI-II* Weschler Abbreviated Scale of Intelligence, *DAS-II* Differential Ability Scales, *VIQ* Verbal IQ (WASI VIQ scale = Verbal Comprehension Index), *NVIQ* Nonverbal IQ (DAS NVIQ scale=Special Nonverbal Composite, WASI NVIQ scale = Perceptual Reasoning Index), *SCQ* Social Communication Questionnaire, *ADOS-2* Autism Diagnostic Observation Schedule – 2nd edition, *CSS* Calibrated Severity Score

**
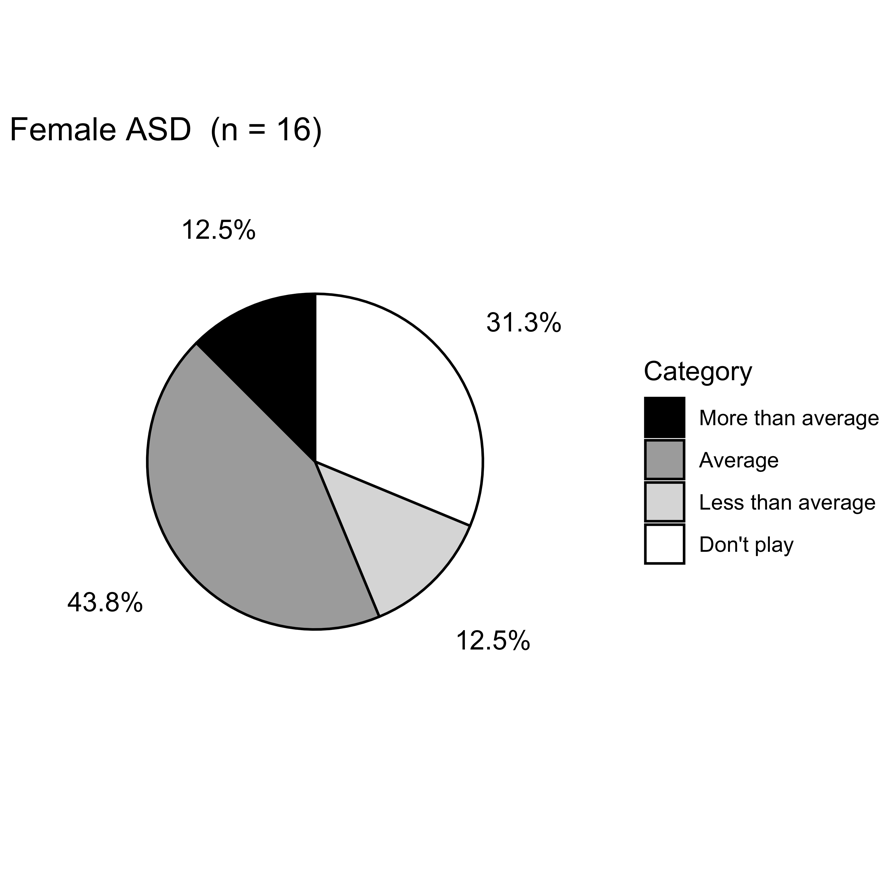

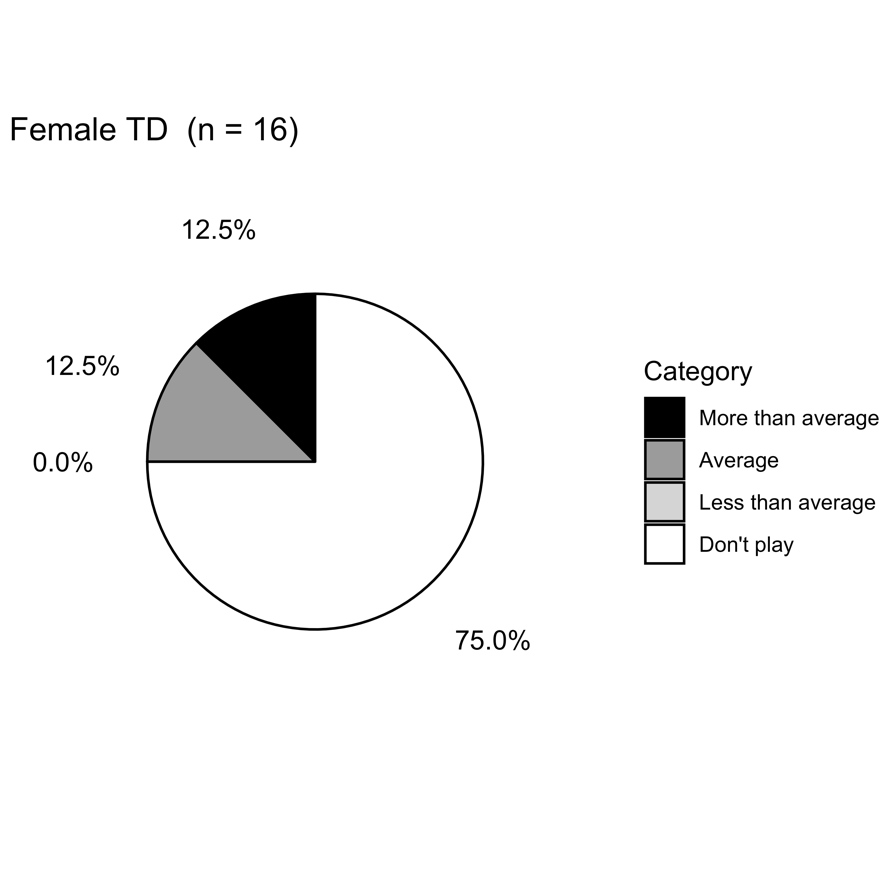
**

**
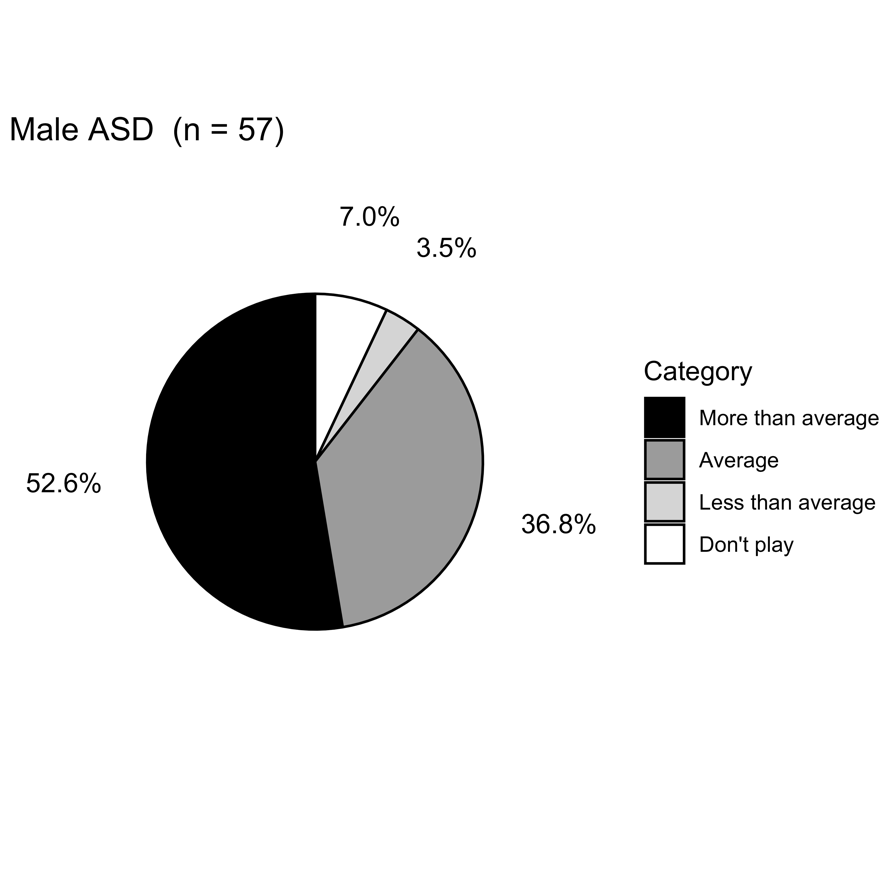

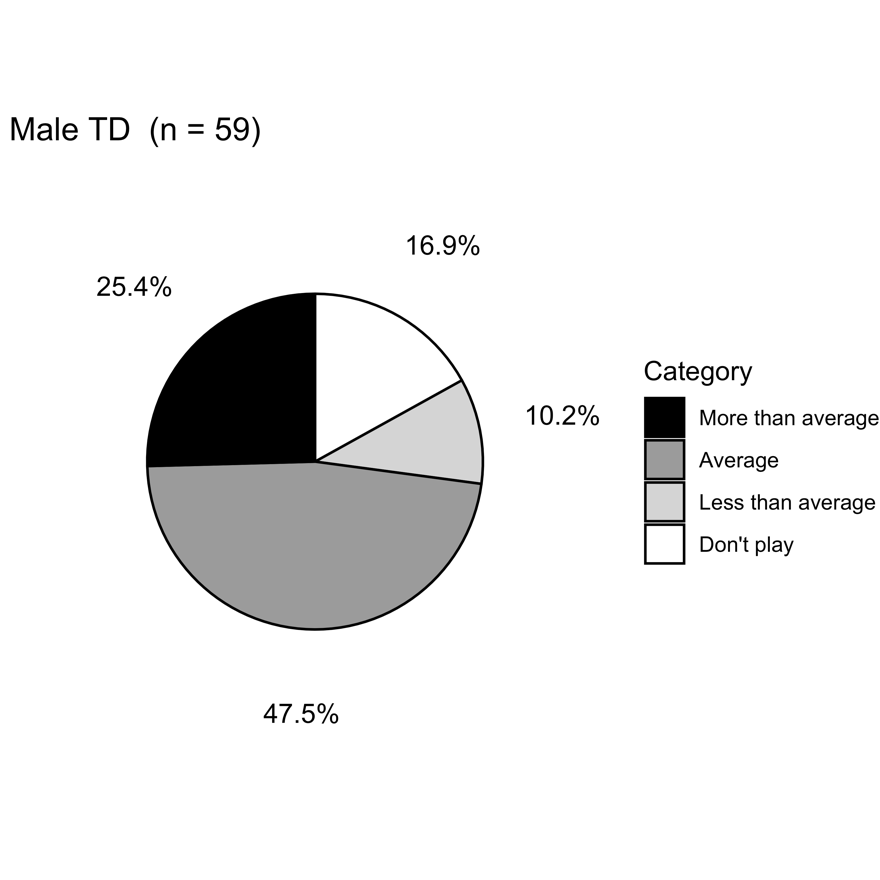
**

**Supplemental Fig. 1** Video game playing category is shown for female and male participants separately. Amount of time spent playing video games, based on caregiver’s CBCL response, was greater for participants with ASD than participants with TD, regardless of sex **(**$\boldsymbol{\chi}^{\boldsymbol{2}}$**(1, *n*=148) = 12.21, *p* < .001)**.

*ASD* Autism Spectrum Disorder, *TD* Typical Development, *CBCL* Child Behavior Checklist

**
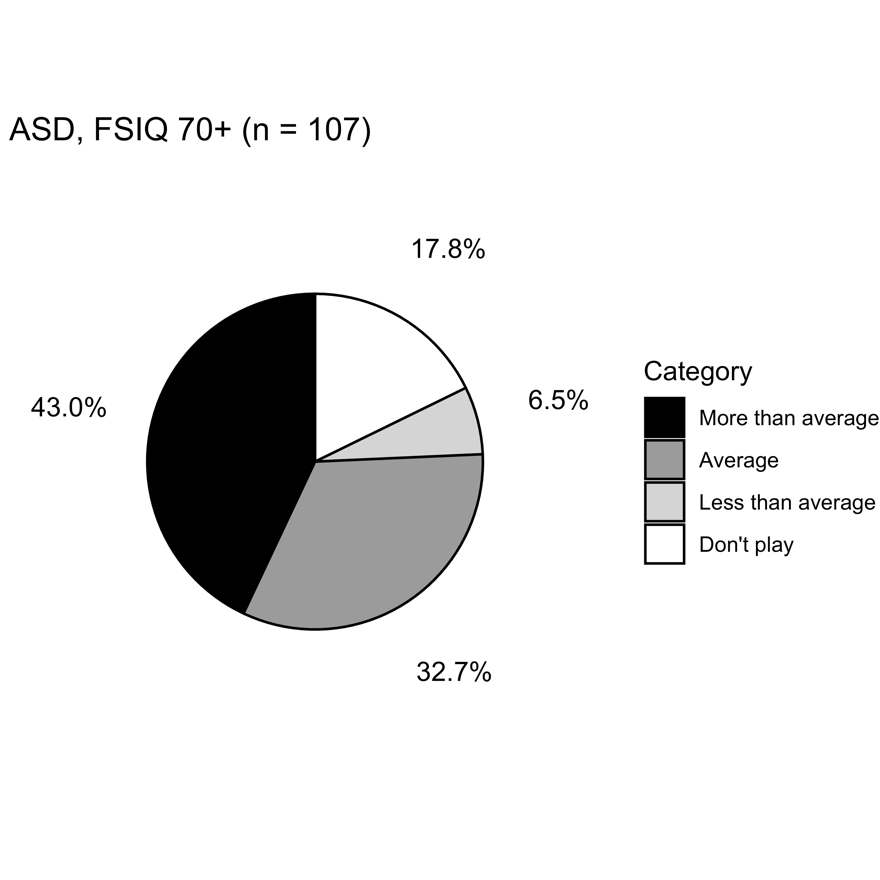

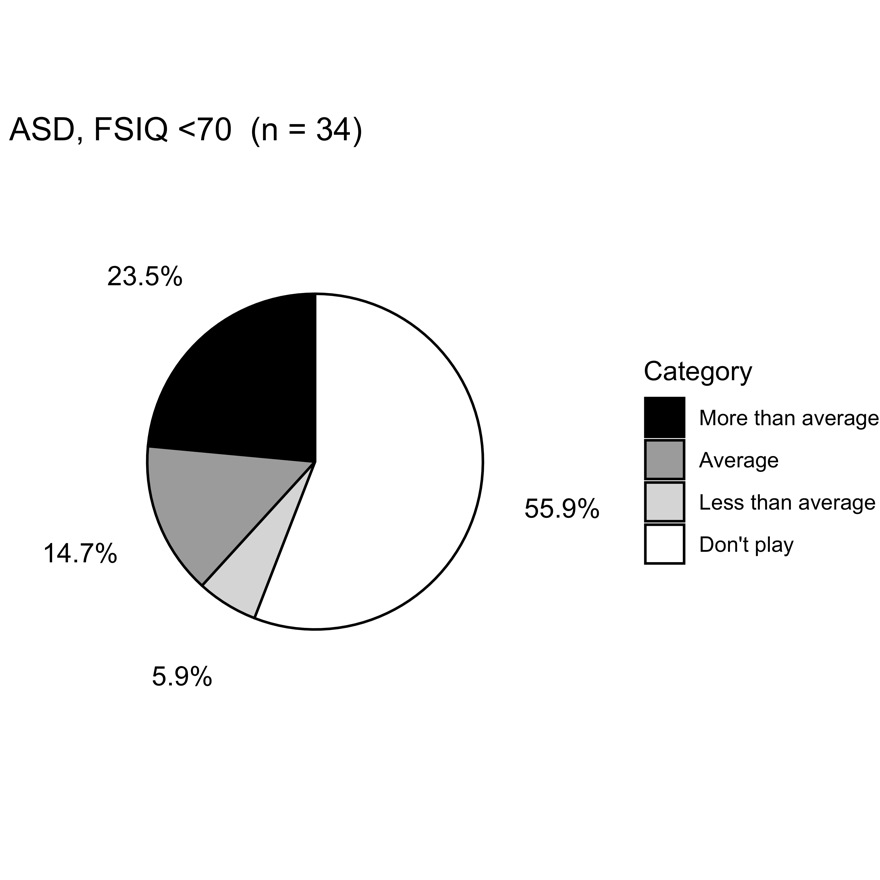
**

**Supplemental Fig. 2** Amount of time spent playing video games, based on caregiver’s CBCL response, was greater for ASD participants with FSIQ ≥ 70 than ASD participants with FSIQ < 70 ($\boldsymbol{\chi}^{\boldsymbol{2}}$**(1, *n* = 141) = 15.68, *p* < .001)**. Results were also significant when comparing video game playing status (Player/Non-Player; $\boldsymbol{\chi}^{\boldsymbol{2}}$**(1, *n*=147) = 18.03, *p* < .001).**

*ASD* Autism Spectrum Disorder, *FSIQ* Full-scale IQ, *CBCL* Child Behavior Checklist
